# Gilbert or Crigler–Najjar syndrome? Neonatal severe unconjugated hyperbilirubinemia with P364L UGT1A1 homozygosity

**DOI:** 10.1186/s13052-022-01251-4

**Published:** 2022-04-18

**Authors:** Laura Cozzi, Federica Nuti, Irene Degrassi, Daniela Civeriati, Giulia Paolella, Gabriella Nebbia

**Affiliations:** 1 Università Degli Studi Di Milano, Milan, Italy; 2 Fondazione IRCCS Ca’ Granda Ospedale Maggiore Policlinico, Pediatric Liver Unit, Milan, Italy; 3 Department of Pediatrics, Vittore Buzzi Children’s Hospital, Milan, Italy

**Keywords:** p.Pro364Leu, UGT1A1, Neonatal severe unconjugated hyperbilirubinemia, Gilbert syndrome, Crigler-Najjar syndrome, Case report

## Abstract

**Background:**

Several mutations of bilirubin uridine diphosphate-glucuronosyltransferase gene (UGT1A1) have been reported in patients with unconjugated hyperbilirubinemia. Few reports are available about the p.Pro364Leu mutation (P364L, c.1091C > T) in homozygous newborns. We describe the clinical, laboratory and therapeutic approach in two Chinese neonates with severe jaundice, homozygous for the P364L mutation.

**Case presentation:**

Two Chinese breastfed female infants presented prolonged unconjugated hyperbilirubinemia at the age of 1 month. Total bilirubin was higher than 15 mg/dl (D < 1). An exhaustive etiological work-up to detect possible causes of hyperbilirubinemia (notably hemolytic ones) was negative.

The promoter and coding regions of UGT1A1 were amplified by polymerase chain reaction (PCR) from genomic DNA isolated from leukocytes. Both patients resulted homozygous for a variant site within the coding region of the gene in the 4 exon, c.1091C > T, p.Pro364Leu.

In front of the persistently high level of unconjugated bilirubin, phototherapy was performed without persistent results. A treatment with phenobarbital was then begun and bilirubin level progressively decreased, with a complete and persistent normalization. The therapy was stopped.

**Conclusion:**

UGT1A1 enzyme activity associated with the P364L mutation has been described as 35.6% of the wild-type enzyme activity. Photo-therapy and phenobarbital can be useful in front of persistently high level of unconjugated bilirubin.

Our cases presented high bilirubin values, overlapping between Gilbert syndrome (GS) and Crigler-Najjar syndrome type II (CNS), but the complete normalization of bilirubin makes GS more likely. Homozygous P364L variant can be associated with severe neonatal unconjugated hyperbilirubinemia in Chinese infants, but jaundice can completely resolve in a few months, contrary to what happens in Crigler-Najjar syndrome type II.

## Introduction

Several mutations of the bilirubin uridine-diphosphate-glucuronosyltransferase gene (UGT1A1) have been reported in patients with unconjugated hyperbilirubinemia. They can be detected both in the promoter or in the coding region and they have been associated with Gilbert syndrome (GS—OMIM 143,500), Crigler-Najjar syndrome type II (CNS II -OMIM 606,785) and type I (OMIM 218,800), with a progressive increase in severity.

The UGT1A1 mutations associated with Gilbert syndrome appear to be considerably different among ethnic groups [1, 2]. The most frequent one in the Caucasian population is A(TA)7TAA(c.-40_-39dupTA or c.-40_-39insTA)(with about 30–50% of residual enzyme activity) [2, 3]. In Asian people G71R(c.211G > A) polymorphism is very frequent, with a 40–70% decrease in UGT1A1 enzyme activity [2, 3]. The G71R variant has been variably associated not only with GS, but occasionally with CNS II [4–6]. Reports are contradictory and the correlation is very difficult to define, because the cohorts of homozygous patients are quite rare. Furthermore, other factors that could contribute to the patient’s bilirubin levels, such as gestational age, age of the patient [7], breastfeeding, epigenetic modifiers, miRNAs and polymorphisms [4–6, 8], have to be considered when evaluating the association between a rare variant of UGT1A1 gene and Gilbert or CNS II syndrome.

In the early 2000s, a novel missense variant P364L (c.1091C > T, p.Pro364Leu) was identified in Asian patients with GS [1, 2]. It was frequently described in heterozygosity [2–4]. This mutation is responsible for the elevation of serum total bilirubin, as shown in COS-7 cells transfected with P364L-cDNA inserted vector [2]. The UGT1A1 enzyme activity associated with the mutation has been described as 64.4% lower than the wild-type enzyme activity [2, 9]. The mutation was at first identified in a heterozygous GS patient, but then it was associated both with “prolonged unconjugated hyperbilirubinemia” [3] and with CNS II (with the G71R variant or the promoter variant c.-40_-39insTA) [10]. Many studies describe patients carrying heterozygous P364L plus multiple pathological variants, resulting in high serum bilirubin levels, and in these cases the contribution of the single variant is not clear [5, 10, 11].

We describe two neonates homozygous for the P364L variant who presented prolonged severe unconjugated hyperbilirubinemia.

## Case presentation

Two Chinese, not related, breastfed female infants presented prolonged unconjugated hyperbilirubinemia at the age of 1 month. They were born respectively at 38th and 39th week of gestational age (from vaginal delivery), with adequate weight, length and head circumference. Both children were born after a normal pregnancy, with an uneventful labour, a normal adaptation, and were discharged from hospital in a few days, without the need of phototherapy.

At one month severe jaundice was still present: total bilirubin of 21.3 mg/dl (direct bilirubin -DB- 0.52) and 16.3 mg/dl(DB 0.56) respectively.

The babies were asymptomatic, without hepatosplenomegaly or vomiting.

An exhaustive etiological work-up to detect possible causes of hyperbilirubinemia (notably hemolytic causes) was negative. The two newborns had normochromic urine and stool, they were growing regularly, and had normal values of AST, ALT, GGT, alkaline phosphatase, C reactive protein, complete blood count. ENS (Extended Neonatal Screening) and urine exam were normal. Both children had normal thyroid function and there was no family history of thyroid disease. They had no siblings, no family history of hemolytic or hepatic diseases and TORCH screen was negative. The abdominal ultrasound was normal and no pyloric stenosis was present.

Both babies underwent phototerapy.

**Fig. 1:**
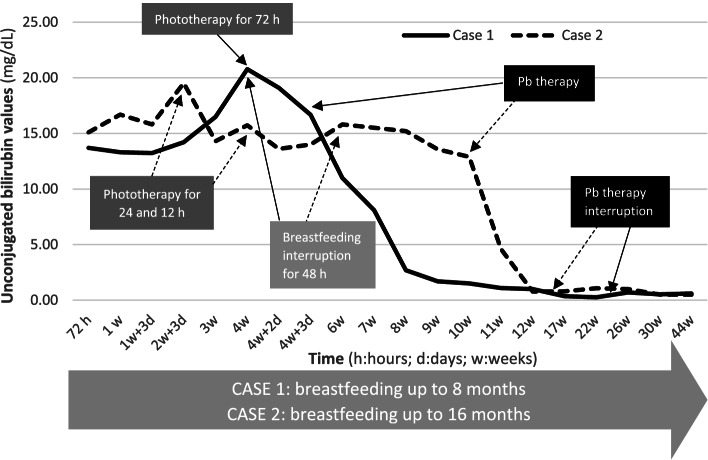
Evolution of unconjugated bilirubin values with different therapies in the two reported cases

**Fig. 2:**
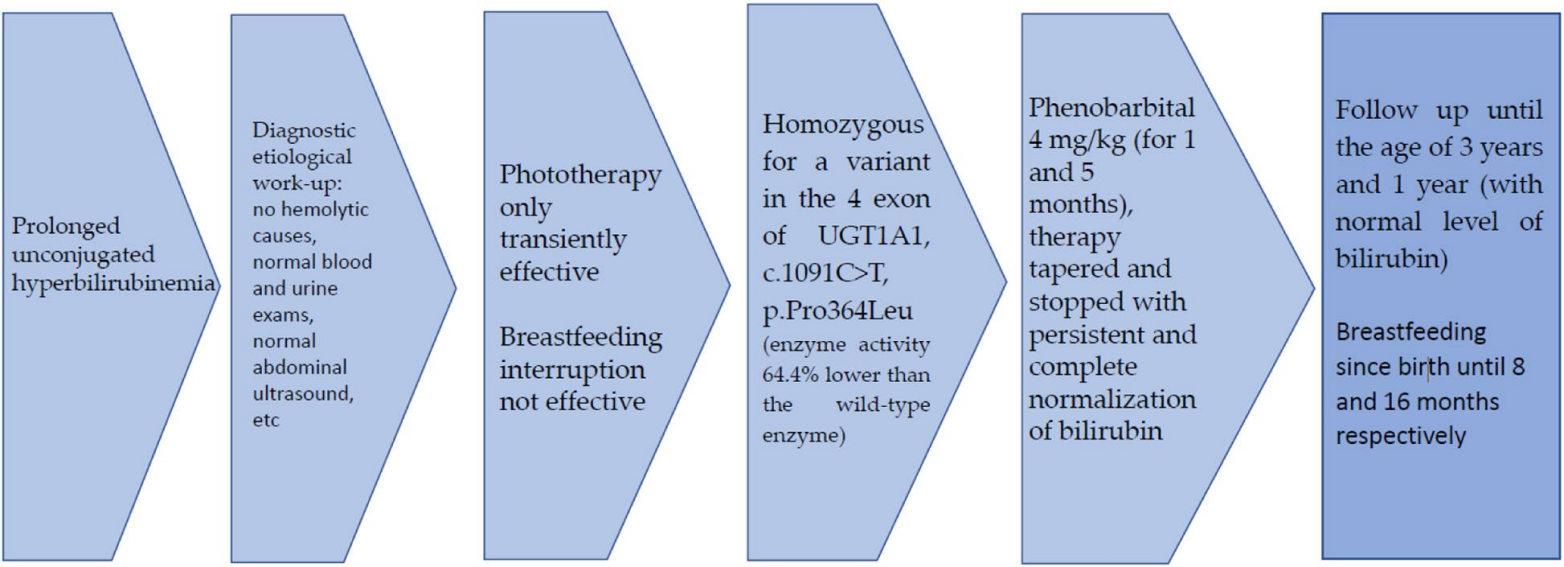
Timeline

Phototherapy was only partially effective, as bilirubin decreased, but not in a significant and persistent way, thus phenobarbital was added in the suspicion of CNS II. Facing the persistent, severe jaundice, the children were treated with phenobarbital at the daily dosage of 4 mg/kg and genetic study of UGT1A1 gene was performed: the promoter and coding regions were amplified by polymerase chain reaction (PCR) from genomic DNA isolated from leukocytes. The children were treated respectively for 5 and 1 month and a complete normalization of the bilirubin values was reached (Figs. 1 and 2); therapy was thus progressively tapered and stopped. The genetic testing showed that both patients were homozygous for a variant site within the coding region of the gene in the 4 exon, c.1091C > T, p.Pro364Leu. The G71R(c.211G > A) polymorphism or the A(TA)7TAA(c.-40_-39dupTA or c.-40_-39insTA) variation in UGT1A1 promoter were not present.

The babies were breastfed up to 16 and 8 months respectively. After the discontinuation of phenobarbital, the children were followed up to the age of 3 years and 1 year respectively, with persistently normal levels of serum bilirubin.

## Discussion and conclusions

Variations in the bilirubin UGT1A1 gene can induce different degrees of reduction in enzyme activity, resulting in unconjugated hyperbilirubinemia. The distinction between Crigler-Najjar syndrome type I or type II and GS is based also on serum bilirubin levels. These clinical entities are considered to be the spectrum of a single disorder with different grades of severity [3, 11, 12].

Our cases presented bilirubin values overlapping between GS and CNS type II, but the complete normalization of bilirubin makes GS more likely [2, 12]. In fact, in CNS type II no normalization of bilirubin is usually observed, owing to the severe lack of enzyme activity (usually < 10%) [5]. CNS type I (absence of the enzyme) was excluded on clinical and laboratory findings [12].

Even though in GS bilirubin amount usually ranges between 1 and 4 mg/dl [12], in some newborns higher values are described, above all when in association with other conditions such as G6PDH, pyloric stenosis or thalassemia [4, 9, 12]. In our patients no other associated factors were found, aside from breastfeeding.

Breastfeeding jaundice is a well-known entity, but its pathogenesis is still unclear [12, 13]. The initial hypothesis concerned the presence of substances capable of interfacing with hepatic conjugation in mother's milk (such as pregnanediol or an excess of non-esterified free fatty acids) [14]. Another hypothesis concerns the beta-glucuronidase enzyme, which is normally present in the brush border of the intestine and which is able to deconjugate bilirubin, increasing its reabsorption in the enterohepatic circulation [15]. Some studies have shown increased beta glucuronidase activity in breastfed infants compared to formula-fed ones [15]. Other studies focus on the presence of genetic variants of UGT1A1 that appear to be better inhibited by pregnanediol [16]. Further studies evaluated the presence of Gilbert syndrome mutation in breastfed jaundiced infants versus breastfed babies without jaundice and found no differences [12]. In conclusion, the combined effect of UGT 1A1 mutation together with the presence of breast milk may lead to a more severe jaundice in certain infants with predisposing factors [13]. Although prolonged unconjugated hyperbilirubinemia has been reported in formula-fed infants carrying UGT1A1 mutations, breastfed infants are more likely to present with this clinical picture [13]. Nevertheless, not all breastfed infants carrying UGT 1A1 mutations exhibit breast milk jaundice [7, 13]. In our cases breastfeeding jaundice was a possible explanation of the clinical picture, but a brief interruption of breastfeeding was not effective (Fig. 1).

The most severe phenotype of hyperbilirubinemia linked to UGT1A1 mutations is Crigler-Najjar syndrome type I, where the mutations result in inability to synthetize this enzyme. These patients will require lifelong phototherapy treatment until more definitive therapy, such as liver transplant, is proposed [11]. Because in Crigler-Najjar type II some UGT 1A1 activity is preserved, this disease has a more indolent course. The usual therapy for CNS II is phenobarbital(PB), which increases expression of the UGT1A1 gene in the liver and partially decreases the levels of bilirubin [11]. Gene therapy will probably be the final solution for CNS: animal models have been studied [17] and two trials on pediatric patients are ongoing (NCT03223194, NCT03466463).

PB response activity is delineated to a 290-bp distal enhancer module sequence (-3483/-3194) of the UGT1A1 gene called glucuronosyltransferase phenobarbital response enhancing motif (gtPBREM). Human constitutive active nuclear receptor (hCAR) is involved in activation of gtPBREM [18]. PB treatment results into translocation of cytoplasmic receptors like CAR into the nucleus; where it binds to retinoid X receptor and forms a heterodimer, which leads to activation of PB response enhancer element [18]. PB also stimulates hyperplasia of the endoplasmic reticulum (where UGT1A1 is located) [18]. Several studies on co-transfected HepG2 cells and mouse primary hepatocytes offered an excellent model for the examination of the responsiveness of the UGT1A1 to PB [11].

No specific treatment is usually necessary for GS, considering the benignity of the condition, although phenobarbital lowers serum bilirubin levels also in these patients [12]. In our cases phenobarbital apparently reduced bilirubin levels, but they maintained normal bilirubin levels also after the treatment. For this reason, even though the bilirubin levels showed a possible overlap with those described in CNS type II, we considered the cases as affected by an infantile and inducible phenotype of Gilbert’s Syndrome, homozygous for P364L mutation. The normalization of bilirubin probably was also obtained by the increase of UGT1A1 activity usually reached between the age of 6–12 weeks [7].

In other Chinese infants with unconjugated bilirubin level of ≥ 20 mg/dl the P364L variant was described (but in heterozygosity) [6, 10]. Hyperbilirubinemia is common in Chinese people and bilirubin levels are described as higher than in Caucasians, including newborns [1, 10]. Previous studies involving the Chinese population calculated the P364L carrier rate as 1.67% [2, 3] P364L polymorphism is presumably present in a certain percentage of Chinese newborns with severe jaundice.

In conclusion, homozygous P364L variant can be associated with severe neonatal unconjugated hyperbilirubinemia in Chinese infants, but jaundice can completely resolve, unlike what happens in CNS type II.

When evaluating a prolonged and severe neonatal jaundice, especially in Asiatic patients, clinicians must consider the possibility of the presence of P364L mutation in order to make an appropriate diagnosis and prognosis.

## Data Availability

The datasets used and/or analysed during the current study are available from the corresponding author on reasonable request.

## Abbreviations

UGT1A1: bilirubin uridine diphosphate-glucuronosyltransferase gene
P364L: p.Pro364Leu mutation
PCR: polymerase chain reaction
GS: Gilbert syndrome
CNS: Crigler-Najjar syndrome
DB: direct bilirubin
ENS: Extended Neonatal Screening
gtPBREM: glucuronosyltransferase phenobarbital response enhancing motif
hCAR: human constitutive active nuclear receptor
